# Timing of a laparoscopic cholecystectomy in acute moderately severe to severe biliary pancreatitis

**DOI:** 10.1016/j.sopen.2026.05.008

**Published:** 2026-06-11

**Authors:** Sjaak Pouwels, Omar Thaher, Dirk Bausch, Gauri Chillarge, Marcello Di Martino, Stijn van Laarhoven, Miljana Vladimirov, Suhaib J.S. Ahmad, Jens Hoeppner, Chetan Parmar

**Affiliations:** a Department of Surgery, Bielefeld University, Medical School and University Medical Center OWL, Campus Hospital Lippe, Detmold, Germany; b Department of Surgical Oncology, Transplant Surgery and General Surgery, Medical University Gdansk, Gdansk, Poland; c Department of Surgery, Marien Hospital Herne, University Hospital of Ruhr University Bochum, Herne, NRW, Germany; d Department of General Surgery, Cambridge University Hospitals NHS Foundation Trust, Cambridge, United Kingdom; e Department of Health Sciences, University of Piemonte Orientale, Novara, Italy; f Division of Surgery, University Maggiore Hospital della Carità, Novara, Italy; g Department of Hepato-Pancreato-Biliary (HPB) and Transplant Surgery, Leiden University Medical Center, Leiden, the Netherlands; h Department of General Surgery, Betsi Cadwaladr University Health Board, Wales, United Kingdom; i Department of Emergency Medicine, Inselspital University of Bern, Bern, Switzerland; j Department of Surgery, Whittington Hospital, London, United Kingdom; k Apollo Hospitals Research and Education Foundation, India; l University College London, UK

**Keywords:** Laparoscopic cholecystectomy, Biliairy pancreatitis, Complications, Mortality, Timing, HPB surgery

Dear editor,

Acute pancreatitis (AP) is one of the most common gastrointestinal disorders, which has a variety of causes including gallstones and/or biliary sludge [1], [2]. The vast majority of the patients have a mild to moderate form of disease [3]. To reduce the risk of recurrent gallstone related complications, like (recurrent) pancreatitis, cholecystitis, cholangitis or gallstone colics, a timely cholecystectomy is indicated in these patients [4], [5], [6].

In the last few years, several aspects of the definitive treatment of the biliary pancreatitis (BP) have changed, based on some pivotal trials [7], [8]. It was seen that approximately one-third of the patients with a BP in the United Kingdom did not receive any definitive treatment within one year after discharge from the hospital [9], [10]. This is conflicting with the recommendations from several guidelines indicated a cholecystectomy during the same admission or at least in 2–6 weeks after discharge from the hospital [4], [5]. Main reasons for the delay of a cholecystectomy was the perceived danger of potential complications, like anatomical problems due to inflammation and oedema and increased risk for conversion [9], [10]. The recent multicentre randomised PONCHO trial showed that same-admission cholecystectomy reduces the risk of recurrent gallstone related complications without increasing the difficulty of surgery in patients with a mild BP [8]. They also concluded that same-admission cholecystectomy (SAC) in patients with BP has a very low cholecystectomy complication rate [8]. The study group also investigated cost-effectiveness in this approach. They showed that a same-admission cholecystectomy was superior to an interval cholecystectomy (IC), with a societal incremental cost-effectiveness ratio of -€1918 to prevent one readmission for gallstone-related complications [7]. The question remains whether this is also the case in patients with a moderately severe biliary pancreatitis (MBP) to severe biliary pancreatitis (SBP).

Current guidelines highlight the lack of consensus on the timing of cholecystectomy in patients with acute MBP or SBP. Current guidelines recommend an early cholecystectomy in patients with a mild acute biliary pancreatitis, based on high level of evidence (1 A). Unfortunately there is still a lack of solid evidence on the timing of cholecystectomy in patients with MBP and SBP. The recent multicentre PONCHO RCT [8] showed that SAC reduced the rate of recurrent gallstone-related complications in patients with mild gallstone acute pancreatitis with a very low risk of post-operative complications, in comparison with DC. Additionally, a post-hoc analysis of the same RCT [7] demonstrated that SAC was less costly than IC/DC.

Regarding MBP and SBP, current guidelines provide contradictory statements what could be recommended in terms of the ‘perfect’ timing for a laparoscopic cholecystectomy. The German Pancreatitis guideline (“S3 Leitlinien Pankreatitis) indicated that the timing of a laparoscopic cholecystectomy in MBP and SBP is difficult to determine and therefore needs to be discussed on a case-by-case basis. The Dutch Acute Pancreatitis guideline follows the guidelines of the International Association of Pancreatology (IAP) / American Pancreatic Association (APA) [4]. In these guidelines there is a clear recommendation that a cholecystectomy should be delayed in patients with peripancreatic collections until the collections resolved or after six weeks. No other recommendations are being done on the timing of cholecystectomy in patients acute SBP [4]. Similar recommendations are being done in the recently updated guidelines for the management of acute pancreatic of the American College of Gastroenterology [6]. They state that any intervention (surgical, radiological and/or endoscopic) in stable patients with pancreatic necrosis or collections should be delayed for at least 4 weeks. No other recommendations are given [6].

Regarding the timing of cholecystectomy there are two major issues that need to be taken into account in patients with MBP and SBP:

1) the potentially increased risk of perioperative complications when early cholecystectomy is performed in patients with MBP and SBP and.
2) the risk of gallstone-related events progressively increases with time if the cholecystectomy is not performed.

What emerges from the available data is that caution should be exercised in routinely recommending EC for patients with MBP and SBP. However, the data on MBP remain limited, leading to significant gaps in our understanding of its optimal management. Substantial differences in baseline characteristics between study and control groups across the included studies and the lack of specific data on MBP complicate the interpretation of outcomes and comparisons. Especially for patients with SBP, it would be valuable to separately analyse those undergoing EC concurrently with other surgical interventions, such as necrosectomy, versus those undergoing EC alone. Another limitation is the frequent absence of data on inflammatory markers at the time of cholecystectomy. While markers at the initial diagnosis of ABP are typically reported, their progression or resolution during the interval leading up to surgery remains poorly understood. Such information is crucial in assessing whether ongoing inflammatory processes, particularly those involving the hepatic hilum, pose clinical challenges during surgery. Similarly, while increased morbidity and mortality associated with EC have been reported, a more detailed evaluation of specific cholecystectomy-related complications would be insightful. This data would enable a more comprehensive risk assessment and provide a clearer understanding of the trade-offs between the benefits of preventing recurrent gallstone-related events and the risks inherent in performing EC.

## CRediT authorship contribution statement

**Sjaak Pouwels:** Writing – review & editing, Writing – original draft, Visualization, Formal analysis, Data curation, Conceptualization. **Omar Thaher:** Writing – review & editing, Writing – original draft, Visualization, Formal analysis, Data curation, Conceptualization. **Dirk Bausch:** Writing – review & editing, Writing – original draft, Visualization, Formal analysis, Data curation, Conceptualization. **Gauri Chillarge:** Writing – review & editing, Writing – original draft, Visualization, Formal analysis, Data curation, Conceptualization. **Marcello Di Martino:** Writing – review & editing, Writing – original draft, Visualization, Formal analysis, Data curation, Conceptualization. **Stijn van Laarhoven:** Writing – review & editing, Writing – original draft, Visualization, Formal analysis, Data curation, Conceptualization. **Miljana Vladimirov:** Writing – review & editing, Writing – original draft, Visualization, Formal analysis, Data curation, Conceptualization. **Suhaib J.S. Ahmad:** Writing – review & editing, Writing – original draft, Visualization, Formal analysis, Data curation, Conceptualization. **Jens Hoeppner:** Writing – review & editing, Writing – original draft, Visualization, Formal analysis, Data curation, Conceptualization. **Chetan Parmar:** Writing – review & editing, Writing – original draft, Visualization, Formal analysis, Data curation, Conceptualization.

## Author contributions

**Initial Idea:** SP.

**Literature Search:** SP and GC.

**Data Analysis:** SP and GC.

**Writing and correcting the manuscript:** SP, OT, DB, GC, MdM, SvL, MV, SA, JH and CP.

**Final Approval:** SP, OT, DB, GC, MdM, SvL, MV, SA, JH and CP.

## Funding

None.

## Declaration of competing interest

None.
